# Assessing antiviral treatment efficacy and risk factors for severe COVID-19 in kidney transplant recipients during the Omicron subvariant-dominant period: a retrospective study

**DOI:** 10.1186/s12882-024-03561-7

**Published:** 2024-04-08

**Authors:** Takashi Sakaguchi, Akihiko Mitsuke, Yoichi Osako, Yasutoshi Yamada, Himawari Takeyama, Risako Ogawa, Katsuya Takahashi, Yukiko Hirohata, Sayuri Yamamoto, Junya Arima, Wataru Fukumoto, Satoshi Sugita, Satoru Inoguchi, Ryosuke Matsushita, Hirofumi Yoshino, Shuichi Tatarano, Hideki Enokida

**Affiliations:** Department of Urology, Graduate School of Medical and Dental Sciences, Kagoshima University, 8-35-1 Sakuragaoka, 890-8520 Kagoshima, Japan

**Keywords:** COVID-19, Kidney transplant recipients, Molnupiravir, Remdesivir

## Abstract

**Background:**

Kidney transplant recipients (KTRs) are at risk of severe coronavirus disease 2019 (COVID-19), and even now that Omicron subvariants have become dominant, cases of severe disease are certain to occur. The aims of this retrospective study were to evaluate the efficacy of antiviral treatment for COVID-19 and to identify risk factors for severe disease in KTRs during Omicron subvariant-dominant periods.

**Methods:**

A total of 65 KTRs diagnosed with COVID-19 who received antiviral treatment between July 2022 and September 2023 were analyzed. Mild cases received oral molnupiravir (MP) as outpatient therapy, while moderate or worse cases received intravenous remdesivir (RDV) as inpatient therapy. In principle, mycophenolate mofetil was withdrawn and switched to everolimus. We investigated the efficacy of antiviral treatment and compared the clinical parameters of mild/moderate and severe/critical cases to identify risk factors for severe COVID-19.

**Results:**

Among 65 cases, 49 were mild, 6 were moderate, 9 were severe, and 1 was of critical severity. MP was administered to 57 cases; 49 (86%) improved and 8 (14%) progressed. RDV was administered to 16 cases; 14 (87%) improved and 2 (13%) progressed. Seventeen (26%) cases required hospitalization, and none died. Comparisons of the severe/critical group (*n* = 10) with the mild/moderate group (*n* = 55) demonstrated that the severe/critical group had a significantly higher median age (64 vs. 53 years, respectively; *p* = 0.0252), prevalence of diabetes (70% vs. 22%, respectively; *p* = 0.0047) and overweight/obesity (40% vs. 11%, respectively; *p* = 0.0393), as well as a significantly longer median time from symptom onset to initial antiviral therapy (3 days vs. 1 day, respectively; *p* = 0.0026). Multivariate analysis showed that a longer time from symptom onset to initial antiviral treatment was an independent risk factor for severe COVID-19 (*p* = 0.0196, odds ratio 1.625, 95% confidence interval 1.081–2.441).

**Conclusion:**

These findings suggest that a longer time from symptom onset to initial antiviral treatment is associated with a higher risk of severe COVID-19 in KTRs. Initiating antiviral treatment as early as possible is crucial for preventing severe outcomes; this represents a valuable insight into COVID-19 management in KTRs.

## Background

Severe acute respiratory syndrome coronavirus 2 (SARS-CoV-2), the virus that causes coronavirus disease 2019 (COVID-19), has been a worldwide pandemic virus since early 2020. With each COVID-19 outbreak, new subvariants are created, and to this day, the disease shows no signs of abating. In addition to risk factors such as diabetes mellitus (DM), hypertension (HT), malignancies and cardiovascular disease (CVD), renal disease is considered a risk factor for COVID-19, and dialysis, organ transplantation, and chronic kidney disease have been reported as factors associated with a higher risk of death [1]. Moreover, organ transplant recipients, including kidney transplant recipients (KTRs), who take immunosuppressive agents have been reported to have a higher rate of severe COVID-19, as well as a lower rate of antibody acquisition with vaccination [2, 3].

The Omicron subvariants, which became the dominant variants as of 2023, have increased the number of infections due to strong immune escape, even in vaccinated people, while the rates of severe illness and mortality have decreased [4]. At our institution, the number of KTRs with COVID-19 increased during the Omicron subvariant era. Although most cases are mild, some KTRs develop severe disease.

Several antiviral drugs, such as molnupiravir (MP), nirmatrelvir/ritonavir (NR), remdesivir (RDV), and ensitrervir, are used to treat COVID-19. MP is an orally available prodrug with antiviral activity against SARS-CoV-2. A Phase III, double-blind, randomized, placebo-controlled study showed that early treatment with MP reduced the risk of hospitalization and death in unvaccinated COVID-19 patients at risk of severe disease [5]. In addition, MP has been shown not to be a substrate, inhibitor, or inducer of various cytochrome P450 (CYP) enzymes, human P-glycoprotein, or evaluated transport proteins, making it a drug with a low risk of drug interactions, which thus may be easier to use in KTRs who are taking a variety of drugs, including immunosuppressive agents [6]. RDV is another antiviral drug that was effective against COVID-19 in several large clinical trials in patients with mild to severe disease [7]. Therefore, we treat KTRs with mild COVID-19 with MP and KTRs with moderate or worse disease with RDV. However, reports regarding the efficacy of antiviral treatment for COVID-19 occurring in KTRs are anecdotal, providing insufficient evidence [8–11]. In addition, risk factors for severe disease in KTRs with COVID-19 during Omicron subvariant- dominant periods are not yet known. The aim of this retrospective study was to evaluate the efficacy of antiviral treatment for COVID-19 and risk factors for its severity in KTRs during Omicron subvariant-dominant periods.

## Patients and methods

### Patient selection

We performed a retrospective observational study at the Department of Urology, Kagoshima University Hospital in Kagoshima, Japan. We obtained informed consent from all subjects and/or their legal guardians in the form of opt-out on the website and permission from the Ethics Committee of Kagoshima University to conduct this study in accordance with the Declaration of Helsinki. A total of 65 Japanese adult KTRs diagnosed with COVID-19 and treated with antiviral agents in our department or related facilities from July 2022 to September 2023 were analyzed. Only symptomatic patients were considered for antiviral treatment in this study. In our institution, the use of antiviral drugs is not indicated in KTRs or patients with other risk factors for severe COVID-19, such as DM and obesity, if they are asymptomatic or have no significant clinical symptoms. As part of their rationale, Sakurai et al. reported on the natural history of asymptomatic SARS-CoV-2-infected patients and found that the majority of the cohort (including patients with pre-existing DM and HT) remained asymptomatic [12]. Therefore, asymptomatic patients and those with very mild symptoms were excluded in this study. The diagnosis of COVID-19 was defined as a positive SARS-COV-2 antigen or polymerase chain reaction test of nasal or pharyngeal swab specimens. The severity of COVID-19 was classified from mild to critical according to the National Institutes of Health (NIH) COVID-19 Treatment Guidelines (https://www.covid19treatmentguidelines.nih.gov/): mild, patients with varied symptoms (e.g., fever, cough, sore throat, malaise, headache, muscle pain) but no shortness of breath, dyspnea, or abnormal imaging; moderate, patients with SpO2 ≥ 94% and lower respiratory disease evidenced by clinical assessment or imaging; severe, patients with SpO2 < 94%, PaO2/FiO2 < 300, respiratory rate > 30 breaths/min, or lung infiltrates > 50%; or critical, patients with respiratory failure, septic shock, and/or multiorgan dysfunction.

### Patient management

Patients initially diagnosed with mild disease were treated orally with MP 1600 mg/day for 5 days in a non-hospitalized setting. Patients who progressed to moderate disease or worse despite treatment with MP were hospitalized and switched to treatment with RDV. Moderate or worse cases at initial diagnosis were hospitalized and treated with intravenous RDV. Typically, RDV was administered at a dose of 200 mg/day on the first day, followed by 100 mg/day for 4 days and then up to 10 days, depending on the disease state. Regarding the adjustment of immunosuppressive agents, in patients taking mycophenolate mofetil (MMF), MMF was temporarily withdrawn or reduced and switched to everolimus (EVR). This conversion was attempted based on the results reported by Modelli de Andrade et al. [13]. As a rule, calcineurin inhibitor (CNI) treatment was maintained and, if necessary, the dose was reduced as much as possible according to the trough concentration.

### Data collection

The clinical characteristics of age, gender, medical history, body mass index (BMI), previous SARS-CoV-2 vaccination, comorbidities, antiviral agents, other therapeutic interventions (e.g., steroids, oxygen therapy, changes in immunosuppressive therapy), and baseline estimated glomerular filtration rate (eGFR) were obtained and analyzed. We evaluated the therapeutic efficacy of MP and RDV in terms of reduction in disease severity, hospitalization rate, and incidence of adverse events. For adverse events, acute kidney injury (AKI) was assessed using Kidney Disease Improving Global Outcomes (KDIGO) criteria and other events were assessed using the Common Terminology Criteria for Adverse Events (CTCAE) version 5.0 (https://ctep.cancer.gov/). We also compared clinical parameters between the mild/moderate and severe/critical groups to assess risk factors for severe COVID-19.

### Statistical analyses

The relationships between the two patient groups were analyzed using the Mann–Whitney U test　and Fisher’s exact test. The relationships among three variables were analyzed using Bonferroni-adjusted Mann-Whitney U tests. Univariate analysis was performed to determine variables associated with COVID-19 severity in KTRs. In addition to the variables for which the *p* value was ≤ 0.1 (time from symptom onset to initial antiviral treatment, age, BMI, and DM), sex, MMF use, and renal function, which have been associated with severe COVID-19 in previous reports [1, 14], were covariates in the multivariate analysis. Multivariable analysis was performed with logistic regression. All analyses were carried out using Expert StatView software, version 5.0 (Cary, NC, USA). *P* - values < 0.05 were considered significant.

## Results

### Patient characteristics

Characteristics of all 65 KTRs with COVID-19 who were enrolled in this study are shown in Table 1. The median age was 55 (range 19 − 74) years, 58% of the cohort was male, median time since transplantation was 56 (range 3–162) months, median BMI was 22 (range 16.8–31.8) kg/m^2^ and median baseline eGFR was 45.1 (range 18.3–99.5) mL/min/1.73m^2^. The median number of previous SARS-CoV-2 vaccination doses was 4 (range 0–6), and vaccination coverage was high, at more than 95%. Among the main immunosuppressive drugs, tacrolimus was used in 92% of cases, MMF in 65%, EVR in 52%, and prednisolone in 92%. Regarding comorbidities, DM was present in 29% of cases, HT in 83% of cases, and 15% of cases were defined as overweight/obesity, with BMI > 25 kg/m^2^. The median time from symptom onset to initial antiviral treatment was 1 (range 0–14) day, indicating that the majority of cases received antiviral treatment relatively early after symptom onset.

**Table 1:** Patient characteristics

		Population
Variable		(*n* = 65)
Age, median (range)		55 (19–74)
Gender, n (%)	Male	38 (58)
	Female	27 (42)
BMI, kg/m^2^, median (range)		22 (16.8–31.8)
Time since transplantation, months (range)		56 (3–162)
Donor, n (%)	Living	63 (97)
	Deceased	2 (3)
Previous SARS-CoV-2 vaccine, doses, median (range)		4 (0–6)
	≧ 3 doses, n (%)	59 (91)
	≦ 2 doses, n (%)	5 (7)
	0 dose, n (%)	1 (2)
Time from symptom onset to initial antiviral treatment, days, median (range)		1 (0–14)
Baseline eGFR, ml/min/1.73m^2^, median (range)		45.1 (18.3–99.5)
Primary kidney disease, n (%)	Glomerulonephritis	27 (42)
	DM nephropathy	11 (17)
	Polycystic kidney disease	6 (9)
	Nephrosclerosis	4 (6)
	Others	8 (12)
	Unknown	9 (14)
Maintenance immunosuppression, n (%)	Tacrolimus	60 (92)
	Cyclosporine	7 (11)
	Mycophenolate mofetil	42 (65)
	Everolimus	33 (52)
	Azathioprine	1 (2)
	Prednisolone	60 (92)
	Methylprednisolone	3 (5)
Comorbidities, n (%)	DM	19 (29)
	HT	54 (83)
	Overweight/obesity	10 (15)
	CVD	13 (20)

### Treatment details and outcomes

A summary of disease severity, treatment details, and outcomes is shown in Table 2. Among the 65 cases, 49 (75%) were mild, 6 (9%) moderate, 9 (14%) severe, and 1 (2%) critical in severity. Fever was the most common major symptom, followed by upper respiratory symptoms such as sore throat and cough. Some patients with moderate or severe disease experienced dyspnea. Arthralgia and gastrointestinal symptoms, such as nausea, vomiting, and diarrhea, were also seen in some cases. Seventeen (26%) patients required hospitalization, and 1 (2%) critical patient required intensive care unit (ICU) admission. As antiviral agents, 57 (88%) patients received MP and 16 (25%) received RDV. In terms of drugs used in combination with antiviral treatment, dexamethasone was used in 10 cases (15%), heparin in 5 cases (8%), and baricitinib in 1 case (2%). Oxygen therapy was administered to 10 patients (15%), and ventilator management was administered to 1 (2%) patient with COVIID-19 of critical severity. With regard to immunosuppressive adjustments, MMF withdrawal/reduction was performed in 40 (62%) cases, representing 95% of the cases taking MMF. Only 2 patients who discontinued MMF did not switch to EVR. In addition, CNI withdrawal/reduction was performed in 19 cases (29%) based on trough level. We maintained steroids used as maintenance immunosuppressants and did not add any other steroids for mild/moderate cases (*n* = 55). In contrast, we used dexamethasone 6 mg/day in combination with RDV for 7–10 days for severe/critical cases (*n* = 10), depending on the case. In terms of overall outcome, 48 (74%) patients recovered without hospitalization, and all 17 (26%) patients who required hospitalization were discharged from the hospital with no deaths. These results were considered to be as favorable as those reported previously [10, 15, 16].

**Table 2:** Summary of disease severity, treatment details, and outcomes

		All patients
		(*n* = 65)
Disease severity, n (%)	Mild	49 (75)
	Moderate	6 (9)
	Severe	9 (14)
	Critical	1 (2)
Symptoms, n (%)	Fever	54 (83)
	Sore throat	35 (54)
	Cough	28 (43)
	Nasal mucus	11 (17)
	Headache	11 (17)
	Dyspnea	9 (14)
	Arthralgia	9 (14)
	Dysgeusia	2 (3)
	Nausea or vomiting	2 (3)
	Diarrhea	1 (2)
Hospitalization, n (%)		17 (26)
Intensive care unit admission, n (%)		1 (2)
Antivirals, n (%)	Molnupiravir	57 (88)
	Remdesivir	16 (25)
Other treatment, n (%)	Dexamethasone	10 (15)
	Heparin	5 (8)
	Baricitinib	1 (2)
	Methylprednisolone	1 (2)
	MMF withdrawal/reduction	40 (62)
	CNI withdrawal/reduction	19 (29)
	Oxygen therapy	10 (15)
	Mechanical ventilation	1 (2)
Outcome, n (%)	Without hospitalization	48 (74)
	Discharged	17(26)
	Death	0 (0)

### Outcomes by antiviral drug

**Fig. 1:**
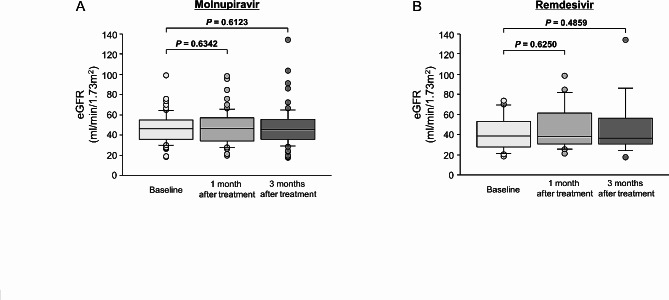
**Comparison of eGFR at baseline, after 1 month of antiviral therapy, and after 3 months of antiviral therapy** Box plot of eGFR at baseline, after 1 month of antiviral therapy, and after 3 months of antiviral therapy. (A) Molnupiravir, (B) Remdesivir

A summary of outcomes by antiviral drug is shown in Table 3. MP was used in 57 patients initially diagnosed with mild disease. By gender, 36 (63%) cases were male and 21 (37%) were female. Of those 57 cases, 49 (86%) were cured and 8 (14%) worsened to moderate or worse and required inpatient treatment with RDV. Only one of the 49 cured patients was hospitalized for AKI. This AKI might have been due to SARS-CoV-2 infection itself, although the possibility of an adverse event with MP could not be ruled out. In fact, there was no significant change from baseline in eGFR at 1 and 3 months after treatment in patients receiving oral MP (Fig. 1A). No other apparent adverse events of concern were observed. RDV was used in 16 patients initially diagnosed with moderate or worse disease. By gender, 7 (44%) cases were male and 9 (56%) were female. All 16 patients who received RDV were hospitalized in case of further severe disease and for careful monitoring of blood concentrations of the CNI for dose adjustment. Among those cases, 13 (87%) cases were cured; two (13%) had disease progression but eventually improved, and all were discharged from the hospital, with no deaths. Adverse events included liver dysfunction (grade 2) in 3 patients and AKI (stage 1) in 1 patient, but none were serious, and all patients improved. Similar to the findings with MP treatment, there was no significant change from baseline in eGFR at 1 month and 3 months after treatment in patients receiving intravenous RDV (Fig. 1B). These results demonstrated the efficacy and safety of MP and RDV for KTRs with COVID-19.

**Table 3:** Outcomes by antiviral drug

		Molnupiravir	Remdesivir
		(*n* = 57)	(*n* = 16)
Age, median (range)		55 (19–74)	62 (27–74)
Gender, n (%)			
	Male	36 (63)	7 (44)
	Female	21 (37)	9 (56)
Hospitalization, n (%)		9 (16)	16 (100)
Disease progression, n (%)		8 (14)	2 (13)
Adverse events, n (%)		1 (2)	4 (25)
	Liver dysfunction ( ≦ G2)	―	3 (19)
	Acute kidney injury (Stage 1)	1 (2)	1 (6)

In addition, we performed a retrospective comparison of the clinical parameters of patients treated with MP who improved and progressed. The results are shown in Table 4. In the group that progressed, the median age was significantly higher than in the group that improved (65.5 vs. 53 years, respectively; *p* = 0.0118), and the time from symptom onset to initial MP treatment was significantly longer (2.5 days vs. 1 day, respectively; *p* = 0.0430). These results suggested the importance of early initiation of MP in mild cases.

**Table 4:** Comparison of clinical parameters of patients treated with MP who improved and progressed

		improvement	progression	P value
		(*n* = 49)	(*n* = 8)	
Age, years, median (range)		53 (19–74)	65.5 (47–74)	0.0118
Gender, n (%)	Male	31 (63)	5 (63)	> 0.9999
	Female	18 (37)	3 (38)	
DM, n (%)		10 (20)	3 (38)	0.3653
BMI, kg/m^2^, median (range)		22 (17.0–29.6)	22.8 (18.8–28.8)	0.2104
	> 25, n (%)	5 (10)	3 (38)	0.0742
Use of MMF as maintenance immunosuppression, n (%)		30 (61)	5 (63)	> 0.9999
Time from symptom onset to MP treatment, days, median (range)		1 (0–5)	2.5 (1–10)	0.0430
Baseline eGFR, ml/min/1.73m^2^, median (range)		46 (19.2–99.5)	44.45 (18.3–73.5)	0.9451
	< 30, n (%)	4 (8)	2 (25)	0.1943
Previous SARS-CoV-2 vaccine, doses, median (range)		4 (2–6)	4 (0–5)	0.8933

### Evaluation of risk factors for disease severity in KTRs with COVID-19

**Fig. 2:**
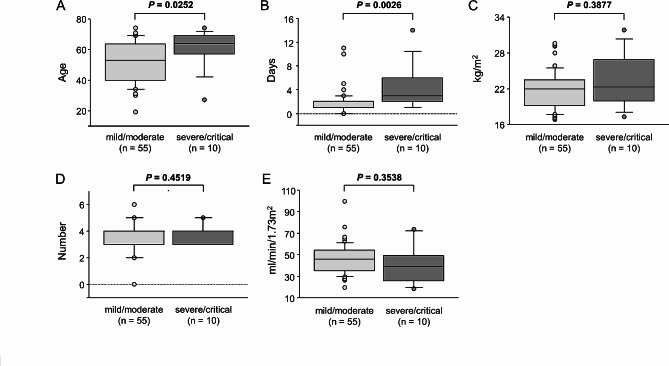
**Comparison of clinical parameters between groups with mild/moderate and severe/critical COVID-19** Box plot of clinical parameters between groups. (A) Age, (B) Time from symptom onset to initial antiviral therapy, (C) BMI, (D) Dose of previous vaccination, (E) eGFR.

To evaluate risk factors for disease severity in KTRs with COVID-19 during the Omicron subvariant-dominant era, we compared the clinical parameters of the mild/moderate (*n* = 55) and severe/critical (*n* = 10) groups. The severe/critical group had a significantly higher median age (64 vs. 53 years, *p* = 0.0252), prevalence of DM (70% vs. 22%, *p* = 0.0047) and prevalence of overweight/obesity (40% vs. 11%, *p* = 0.0393) (Table 5; Fig. 2A). In addition, the time from symptom onset to initial antiviral treatment was significantly longer in the severe/critical group than in the mild/moderate group (3 days vs. 1 day, *p* = 0.0026) (Table 5; Fig. 2B). There were no significant differences in gender, prevalence of HT, median BMI, percentage of patients with MMF use and number of previous vaccinations between groups (Table 5; Fig. 2C-D). On the other hand, although not significantly different, there was a trend toward lower baseline eGFR in the severe/critical group compared with the mild/moderate group and a trend toward a higher proportion of patients with eGFR < 30 ml/min/1.73m^2^ (Table 5; Fig. 2E). These results suggested that earlier antiviral treatment might be important in reducing disease severity, in addition to managing known risk factors for severity, such as age and DM.

**Table 5:** Comparison of baseline characteristics between groups

		mild/moderate	severe/critical	P value
		(*n* = 55)	(*n* = 10)	
Age, years, median (range)		53 (19–74)	64 (27–74)	0.0252
	≧ 60, n (%)	20 (36)	7 (70)	0.0788
Gender, n (%)	Male	34 (62)	4 (40)	0.2968
	Female	21 (38)	6 (60)	
DM, n (%)		12 (22)	7 (70)	0.0047
HT, n (%)		44 (80)	10 (100)	0.1930
BMI, kg/m^2^, median (range)		22 (16.8–29.6)	22.25 (18.8–31.8)	0.3877
	> 25, n (%)	6 (11)	4 (40)	0.0393
Use of MMF as maintenance immunosuppression, n (%)		34 (62)	8 (80)	0.4736
Time from symptom onset to initial antiviral treatment, days, median (range)		1 (0–11)	3 (1–14)	0.0026
Baseline eGFR, ml/min/1.73m^2^, median (range)		46 (19.2–99.5)	39 (18.3–73.5)	0.3538
	< 30, n (%)	6 (11)	3 (30)	0.1345
Previous SARS-CoV-2 vaccine, doses, median (range)		4 (0–6)	4 (3–5)	0.4519

### A longer time from symptom onset to initial antiviral treatment is an independent risk factor for severe COVID-19 in KTRs

**Fig. 3:**
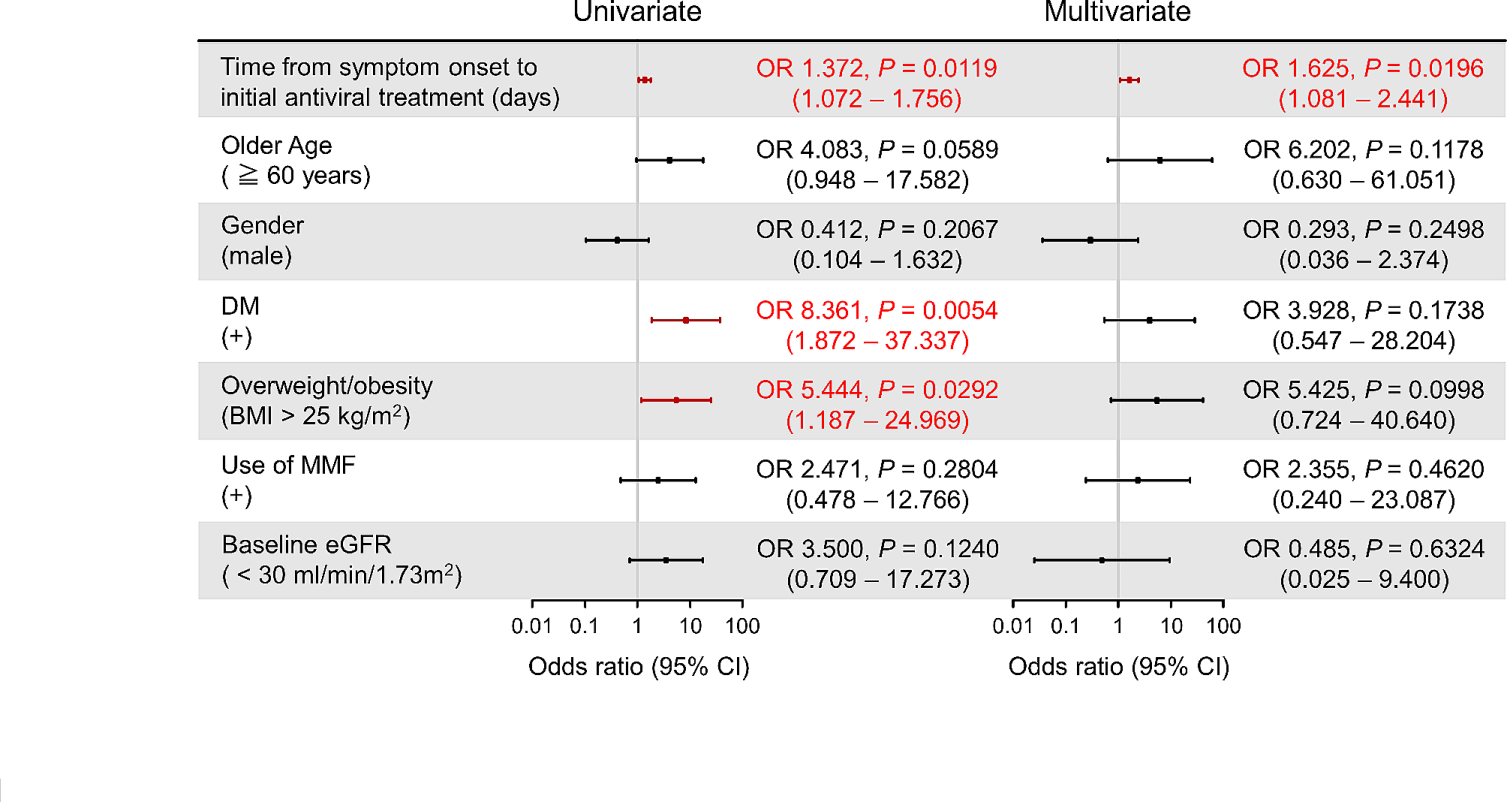
**Univariate and multivariate analysis to evaluate risk factors for severe COVID-19 in KTRs.** Logistic regression analysis showed that longer time from symptom onset to initial antiviral therapy was an independent risk factor for severe COVID-19 in KTRs. OR, odds ratio. CI, confidence interval.

We performed a multivariate logistic regression analysis to investigate independent risk factors for COVID-19 severity in KTRs. Univariate analysis suggested that DM, overweight/obesity and a longer time from symptom onset to initial antiviral treatment were significant risk factors for disease severity (Fig. 3). Of these factors, multivariate analysis identified that longer time from symptom onset to initial antiviral treatment (odds ratio 1.625, 95% confidence interval 1.081–2.441, *p* = 0.0196) was the only independent risk factor for severe COVID-19 in KTRs (Fig. 3). These results provide positive support for the possibility that initiation of antiviral treatment as early as possible might reduce the severity of COVID-19 in KTRs.

## Discussion

More than three years have passed since COVID-19 spread worldwide, but after repeated outbreaks and resurgences, the virulence of this unprecedented infectious disease remains. The widespread use of vaccines and the mutation of SARS-CoV-2 have reduced the rate of severe disease and mortality, but the number of infected patients has not decreased, and there is an urgent need to elucidate the mechanisms of and risk factors for severe disease and sequelae. In addition to their immunosuppressed state, solid organ transplant (SOT) recipients, including KTRs, who often have a variety of comorbidities, are generally considered to be at high risk for severe disease [2]. In a large meta-analysis of patients who became infected with COVID-19 after SOT, including 1500 KTRs, the mortality rate among KTRs was 22% higher than in the general population, and risk factors were similar to those in the general population, such as older age and obesity [17]. COVID-19 severity and mortality rates are reported to be decreased with the Omicron strain. However, Nevejan et al. reported that immunocompromised adult patients showed an increased risk of in-hospital mortality related to COVID-19 when infected with Omicron variants [18]. Moreover, Wong et al. found a mortality rate of 4.9%, multi-organ failure rate of 12.2%, and respiratory failure rate of 12.2% in 41 KTRs with COVID-19, and even in the Omicron dominant period, the authors reported that COVID-19 may not necessarily be mild in KTRs [15]. Similar to the findings of their report, 10 of the 65 cases (15%) in our present study were in the severe/critical group, which does not necessarily mean that the disease can be considered mild.

The effect of chronic immunosuppressive agent use on COVID-19 in KTRs remains unclear. The scientific evidence for conversion of MMF to EVR in KTRs with COVID-19 is still insufficient. Nevertheless, we are converting from MMF to EVR based on the findings of Modelli de Andrade et al. [13]. They analyzed risk factors for mortality in 1379 KTRs in the 28 days following a COVID-19 diagnosis and found that one risk factor for increased mortality was the use of MMF or azathioprine, while the use of mammalian target of rapamycin (mTOR) inhibitors was associated with a decreased risk of death [13]. In their discussion, they hypothesized that the reason for the adverse effects of MMF and azathioprine use is that they are often associated with lymphopenia, a known risk factor for COVID-related death, and that the production of neutralizing antiviral antibodies may be inhibited [13, 19, 20]. In addition, since SARS-CoV-2 replication has been suggested to be dependent on the Akt/mTOR/HIF-1 pathway, the authors hypothesized that the use of mTOR inhibitors might lead to a protective effect [13, 21, 22]. Moreover, there is a report that mTOR inhibitors can inhibit SARS-CoV-2 replication by inducing autophagy and inhibiting viral protein synthesis [23]. Several reports have suggested that switching from MMF to EVR may be effective and safe in SOT recipients with COVID-19 [24–26]. López-Vilella et al. reported that heart transplant recipients with COVID-19 who developed non-severe pneumonia requiring hospitalization were switched from MMF to EVR, which was safe, with no instances of rejection, and was well tolerated [24].. Interestingly, they reported that in this patient population, few (only 8%) received antiviral treatment, but all had good outcomes. Thus, those results suggest that this conversion may be effective and safe in SOT recipients with COVID-19. The investigators also stated that the reason for this conversion was that EVR reduces the risk of infection by other viruses, such as cytomegalovirus, by regulating factors involved in several cellular functions that are crucial for viral replication [24]. In addition, Colmenero et al. proposed an algorithm for changing baseline immunosuppressive agents in liver transplant recipients with COVID-19, based on the fact that MMF dose, especially when higher than 1000 mg/day, is an independent risk factor for severe COVID-19, and this deleterious effect was not observed with EVR or CNIs [25]. In their algorithm, they concluded that MMF withdrawal or temporary conversion to EVR or CNIs until disease resolution could be beneficial for hospitalized patients [25]. Although there are no comprehensive studies involving this conversion in KTRs with COVID-19, Kijima et al. reported a case of COVID-19 in a KTR who had a good outcome after switching from MMF to EVR in addition to antibody cocktail therapy [26]. That case experienced no rejection, stable renal function, and no exacerbation of COVID-19 after conversion, suggesting its efficacy and safety. The authors also explained that one reason for this conversion was that mTOR inhibitors suppress early production of B cells and reduce the population of antigen-specific memory B cells, which would be expected to reduce cross-reactive antibody production and thus antibody-dependent potentiation in patients with COVID-19 [26]. Based on the findings of those studies, in principle, MMF was being withdrawn and actively converted to EVR in our present study. However, despite those previous reports, the scientific basis for this conversion is still inadequate, and several serious concerns about this conversion remain. The first is that it takes several days for EVR to reach steady-state and exert its effect, which may prevent it from having an early effect on COVID-19 in KTRs [27, 28]. Second, EVR may increase the immunosuppressive burden of KTRs with COVID-19. Moreover, mTOR inhibitors may exacerbate the hematologic effects and pneumonia caused by COVID-19 [29]. The fact that these concerns remain unresolved is one of the limitations of this study. Furthermore, this study was not designed to evaluate the effect of any specific treatment on KTRs with COVID-19, and no firm conclusions can be drawn in this regard. Therefore, to resolve these concerns, we would like to accumulate more cases and conduct further research to investigate whether this conversion is appropriate, thereby contributing to a favorable outcome in KTRs with COVID-19.　Regarding the administration of steroids, as mentioned above, dexamethasone was combined with RDV only in severe/critical cases, while mild/moderate cases were maintained on steroids only as maintenance immunosuppressants. Gottlieb et al. reported that the use of dexamethasone in combination with RDV in severe/critical cases decreased mortality while it increased mortality in mild/moderate cases [30]. In addition, Moromizato et al. recently reported that intravenous methylprednisolone pulse therapy for critically ill patients with COVID-19 reduced the risk of in-hospital mortality, whereas it increased the risk of in-hospital mortality in non-critically ill patients [31]. Based on those findings, we administered dexamethasone as an additional steroid only in severe/critical COVID-19 cases. However, there is still no evidence for optimal adjustment of steroids specifically for COVID-19 in KTRs. Therefore, further studies are needed to investigate the appropriate adjustment of steroids for KTRs with COVID-19.

To control the severity of COVID-19 in KTRs, antiviral treatment is very important. Radcliffe et al. reported that SOT recipients treated with MP, sotrovimab or NR and diagnosed with COVID-19 during the Omicron surge period (67% of whom were KTRs) had lower hospitalization and mortality rates compared with untreated patients [16]. This report suggested that there is a significant benefit to antiviral treatment in KTRs with COVID-19, even in the Omicron stage. Although MP has also been reported to contribute to the prevention of post-hospitalization worsening in the Omicron variant phase of COVID-19 [32], few reports have described the benefit of MP for COVID-19 in KTRs. Therefore, the efficacy and safety of MP for COVID-19 in KTRs remains to be investigated. In the aforementioned report by Radcliffe et al., the hospitalization rate for untreated patients with COVID-19 after kidney transplantation was 27%, compared with a rate of 16% for patients treated with MP [16]. In addition, Poznański et al. reported that the hospitalization rate for KTRs with COVID-19 treated with MP was low (12.5%), and no serious side effects or drug interactions with immunosuppressive agents were observed [10]. In our present study, the hospitalization rate for KTRs with COVID-19 treated with MP was 16%, and no serious adverse events were observed, findings that were as favorable as those in the aforementioned reports.

On the other hand, another leading oral drug, NR, has been reported to significantly reduce the risk of hospitalization and death compared with MP [6]. A phase 2/3 double-blind, randomized, controlled trial demonstrated that the risk of progression to severe COVID-19 was 89% lower with NR than with placebo for symptomatic COVID-19, and this result compared favorably with the efficacy of MP [33]. However, nirmatorelvir is a substrate for the P-glycoprotein and CYP3A4 enzymes, and ritonavir is primarily a substrate for the CYP2D6 enzyme as well as CYP3A4, so interactions with many drugs, including immunosuppressants, are a major concern [6, 34]. Although some reports suggest the safety and efficacy of NR in COVID-19 in KTRs, it is essential to implement protocols that adjust for the use of immunosuppressive agents to avoid adverse events due to drug interactions [35]. Based on the above findings, we speculate that the antiviral treatment with MP in only mild cases may have been reasonable in terms of efficacy and avoiding the risk of drug-drug interactions in our present study.

RDV was originally developed as a treatment for Ebola virus infection and its efficacy against COVID-19 has been widely reported. A randomized controlled trial of patients with COVID-19 who required hospitalization in Europe, the U.S., and Asia found a reduction in time to clinical improvement from 15 days in the placebo-treated group to 10 days in the RDV group [36]. It has also been reported that early 3-day treatment with RDV in non-hospitalized patients with COVID-19 who were at risk for severe disease reduced the risk of hospitalization and death by 87% [30]. Several reports have described the efficacy and safety of RDV for KTRs with COVID-19 [9, 37, 38]. Elec et al. reported that ICU mortality was significantly reduced with the use of RDV, and it seemed to have no apparent nephrotoxicity in KTRs [9]. In addition, Solera et al. reported that early RDV administration prevented severe disease due to COVID-19 in SOT recipients (41.7% of whom were KTRs) in Omicron-dominant periods [38]. In our present study, RDV was used in patients with moderate or worse severity, and the efficacy rate was 87%, the ICU admission rate was 2%, and the mortality rate was 0%, which is considered to be as favorable as in previous reports. Besides, RDV is also an inhibitor of CYP3A and is a substrate for P-glycoprotein, so care must be taken to avoid interactions with many drugs, including immunosuppressive agents [39]. We have previously reported drug interactions between RDV and tacrolimus in KTRs with COVID-19, suggesting that blood concentrations of tacrolimus should be monitored and the dose carefully adjusted when administered in combination with RDV [40]. In light of that report, we specified that patients using RDV in this study were to be treated as inpatients and their blood concentrations carefully monitored to avoid serious adverse events. Overall, the efficacy and safety of antiviral treatment with MP and RDV for COVID-19 in KTRs during Omicron subvariant-dominant periods was suggested in this study. Since there are still several problems to be solved, including the selection of appropriate antiviral drugs, the development of new antiviral drugs, and drug interactions with immunosuppressants, it is essential to continue to accumulate more cases and conduct further research.

Vaccination against SARS-CoV-2 is also important with respect to the control of COVID-19 severity. Several previous reports have shown low rates of antibody acquisition and antibody production capacity from vaccination in KTRs [3, 41]. However, Fujieda et al. recently reported that three doses of the SARS-CoV-2 vaccine significantly increased antibody acquisition by 71% in KTRs, suggesting the importance of additional doses in KTRs [42]. The cohort of KTRs with COVID-19 in the current study had a high overall vaccination rate, with more than 90% of patients receiving 3 or more doses of the vaccine. In addition, there was no difference in vaccination status between the mild/moderate group and the severe/critical group. Favorable vaccination status may be one of the reasons why there were no deaths in our study and not as many severe cases as in previous reports.

In terms of risk factors for COVID-19 severity, results of previous studies have shown that older age, obesity, DM and kidney dysfunction are major risk factors for worsening or severe COVID-19 [43]. Based on previous reports, the higher median age and higher rates of DM and overweight/obesity in the severe/critical group were also reasonable findings in our present study. Therefore, in KTRs who have known risk factors for severe COVID-19, such as older age, DM, and obesity, early therapeutic intervention and careful follow-up are necessary, even in mild cases. In this study, there was a tendency for baseline eGFR to be lower in the severe/critical group compared to the mild/moderate group, although the difference was not significant. Moreover, Sato et al. recently investigated whether kidney dysfunction on admission is associated with subsequent severe COVID-19 and increased acute-phase mortality [44]. They reported that kidney dysfunction on admission was significantly associated with the frequency of primary endpoints such as in-hospital death, mechanical ventilation use, and ICU admission [44]. Based on those findings, we believe that efforts to maintain transplant kidney function in daily clinical practice may lead to reducing the severity of COVID-19 infection. On the other hand, SARS-CoV-2 is constantly mutating, and it is therefore considered important to investigate the assessment of risk factors for severe disease at each outbreak. It is interesting to note that in our present study, longer time from symptom onset to initial antiviral treatment was identified as an independent risk factor for severe COVID-19. Recently, Alonso-Navarro et al. reported that early administration of RDV from symptom onset might reduce the risk of ICU admission in patients with COVID-19 requiring hospitalization [45]. Furthermore, Bruno et al. reported that in patients with COVID-19, 77% of whom were treated with MP, early use of oral antivirals improved COVID-19 outcomes in subjects at high risk of disease progression [46]. These previous reports support the findings of the current study, which suggest that early antiviral treatment may be an important factor in reducing the risk of severe disease. To the best of our knowledge, no previous reports have suggested that prolonged time from symptom onset to initial antiviral treatment might be a risk factor for severe COVID-19 in KTRs during the Omicron subvariant-dominant periods, which suggests that our results may be significant. Based on these findings, we believe that it is important to educate not only healthcare providers, but also KTRs themselves in their daily practices, about the importance of initiating antiviral treatment as early as possible after the onset of symptoms of suspected COVID-19, so that there is no delay in the initial therapeutic response.

Limitations of the present study include the small cohort size, the short follow-up period, and the fact that it was a retrospective study. Perhaps in part because of this, the known risk factors for severe disease, such as older age, male sex, DM, and overweight/obesity, were not identified as independent risk factors for severe COVID-19 in the multivariate analysis. It is speculated that the use of MMF, which has been suggested to be a risk factor for severe COVID-19 in KTRs, did not yield significant results in the current study for the same reason. In addition, longer follow-up is needed to assess the effects of COVID-19 and antivirals on renal function and late complications. Furthermore, as mentioned above, a limitation of this study is that there is no scientific evidence to support switching from MMF to EVR, and several concerns remains unresolved. Further long-term accumulation of cases and research is needed to clarify appropriate antiviral treatment, optimal immunosuppressant adjustment, risk factors for severity, and complications in KTRs with COVID-19.

## Conclusion

The results of this retrospective study suggest that a longer time from symptom onset to initial antiviral treatment is a risk factor for severe COVID-19 in KTRs. It was also suggested that in addition to managing known risk factors, such as older age and DM, initiating antiviral treatment as early as possible might be important in preventing severe COVID-19 in KTRs. Because of the small cohort size and short study period of this study, we are extending the study period and accumulating cases to evaluate the long-term prognosis (e.g., long-term effects on transplanted kidney function, late complications) of KTRs with COVID-19. Also, the appropriate adjustment of immunosuppressive agents, such as MMF to EVR conversion and optimal steroid use, is not yet clear. Therefore, we are planning clinical and basic research studies to identify optimal immunosuppressive regimens in KTRs with COVID-19. Further studies are needed to identify appropriate treatment, including optimal immunosuppressant adjustment, for the constantly mutating SARS-CoV-2 and to identify additional risk factors for severe COVID-19 in KTRs.

## Data Availability

The datasets obtained during and/or analyzed during the current study are available from the corresponding author upon reasonable request.

## Abbreviations

KTRs: kidney transplant recipients
COVID-19: coronavirus disease 2019
MP: molnupiravir
RDV: remdesivir
SARS-CoV-2: severe acute respiratory syndrome coronavirus 2
DM: diabetes mellitus
HT: hypertension
CVD: cardiovascular disease
NR: nirmatrelvir/ritonavir
MMF: mycophenolate mofetil
EVR: everolimus
CNI: calcineurin inhibitor
CYP: cytochrome P450
BMI: body mass index
ICU: intensive care unit
eGFR: estimated glomerular filtration rate
AKI: acute kidney injury
SOT: solid organ transplant
mTOR: mammalian target of rapamycin
OR: odds ratio
CI: confidence interval
